# New Insights into the Application of Lithium‐Ion Battery Materials: Selective Extraction of Lithium from Brines via a Rocking‐Chair Lithium‐Ion Battery System

**DOI:** 10.1002/gch2.201700079

**Published:** 2018-01-15

**Authors:** Lihua He, Wenhua Xu, Yunfeng Song, Yunze Luo, Xuheng Liu, Zhongwei Zhao

**Affiliations:** ^1^ School of Metallurgy and Environment Central South University Changsha Hunan 410083 P. R. China; ^2^ Institute of Nuclear and New Energy Technology Tsinghua University Beijing 100084 P. R. China

**Keywords:** brine, LiFePO_4_, lithium extraction, rocking‐chair lithium‐ion batteries

## Abstract

Lithium extraction from high Mg/Li ratio brine is a key technical problem in the world. Based on the principle of rocking‐chair lithium‐ion batteries, cathode material LiFePO_4_ is applied to extract lithium from brine, and a novel lithium‐ion battery system of LiFePO_4_ | NaCl solution | anion‐exchange membrane | brine | FePO_4_ is constructed. In this method, Li^+^ is selectively absorbed from the brine by FePO_4_ (Li^+^ + e + FePO_4_ = LiFePO_4_); meanwhile, Li^+^ is desorbed from LiFePO_4_ (LiFePO_4_ − e = Li^+^ + FePO_4_) and enriched efficiently. To treat a raw brine solution, the Mg/Li ratio decreases from the initial 134.4 in the brine to 1.2 in the obtained anolyte and 83% lithium is extracted. For the treatment of an old brine solution, the Mg/Li ratio decreases from the initial 48.4 in the brine to 0.5 and the concentration of lithium in the anolyte is accumulated about six times (from the initial 0.51 g L^−1^ in the brine to 3.2 g L^−1^ in the anolyte), with the absorption capacity of about 25 mg (Li) g (LiFePO_4_)^−1^. Additionally, it displays a great perspective on the application in light of its high selectively, good cycling performance, effective lithium enrichment, environmental friendliness, low cost, and avoidance of poisonous organic reagents and harmful acid or oxidant.

In recent years, the increasing demands for portable electronic devices, hybrid and plug‐in electric vehicles, and storage of electricity from wind/solar energy promote the development of Li‐ion batteries. Meanwhile, the production of lithium and its compounds for lithium‐ion batteries has attracted more and more attention in the global lithium industry.^1^, ^2^, ^3^ Salt lake brines are considered as valuable resources having great potential for the development of the lithium industry in the world,^4^ and various methods such as chemical precipitation,^1^, ^5^ solvent extraction,^6^ and membrane separation^7^ have been developed for lithium extraction from brines. Nevertheless, the recovery of lithium from a high Mg/Li ratio brine has been a key technical problem in the world due to the similar chemical properties of Mg^2+^ and Li^+^.^5^ Especially in some areas of the world, such as the Qinghai region in China, salt lakes exhibit a characteristically high mass ratio of Mg/Li (reaches 40–200:1 and may be greater than 1800:1 in some cases).^8^ As another example, Uyuni Salar brine as the largest reserves of lithium lake on earth, has still not been economically developed on a large scale due to its high Mg/Li ratio (Mg/Li = 18–22:1).^9^

Ion‐sieve absorption method has been considered to be one of the promising approaches for lithium extraction from the high Mg/Li ratio brines since its high selectivity, low cost, and nontoxicity. Spinel‐type manganese oxide λ‐MnO_2_ as one of the typical Li‐ion sieves, which are generally prepared by using lithium manganese oxides LiMn_2_O_4_, was widely used to extract lithium from the salt lake brines and seawater.^10^ In the absorption process, Li^+^ will insert into λ‐MnO_2_ to reform Li_*x*_Mn_2_O_4_ (0 < *x* < 1), and in the desorption process, Li^+^ will be extracted from Li*_x_*Mn_2_O_4_ with an acid or an oxidizing agent, corresponding with the reformation of λ‐MnO_2_. However, the ion‐sieve absorption method suffers from: (1) difficult preparation of the high absorption capacity absorbent; (2) serious capacity loss in the desorption process due to the use of acid or oxidizing agent as the desorption reagent; and (3) great challenges of absorbent granulation. All of these problems seriously prevent its use on a large scale.

Actually, LiMn_2_O_4_ is not only an Li‐ion sieve material but also an important cathode material that is widely used in rechargeable lithium‐ion batteries.^11^ During the charge–discharge cycles of batteries, lithium ion moves back and forth between the anode and the cathode. This process is similar to a rocking chair, so this system is termed as “rocking‐chair battery.”^12^ Obviously, the behavior of Li^+^ intercalation/de‐intercalation into/from the electrode materials in charge–discharge cycle can also be considered as an alternative lithium absorption/desorption process. The difference is that the traditional desorption is carried out by an acid or an oxidant, but in the battery cell, it is operated by an external circuit. But the cycling performance of LiMn_2_O_4_ battery is much more excellent than that of the traditional absorption process. So, if we use the brines instead of the lithium electrolyte, it is possible to develop a new method for lithium extraction based on this battery's principle.

In theory, any cathode or anode material that can be used in rechargeable lithium‐ion batteries can possibly be used for the lithium extraction from brines. **Figure** **1** and Tang et al^14^ show the main electrode materials that can be used for aqueous lithium‐ion batteries. Taking into consideration the stability, easy preparation, low cost, and environmentally friendly of LiFePO_4_, it can be chosen as the electrode materials for lithium recovery. More importantly, recent research studies have verified that LiFePO_4_ has a great cycling performance in aqueous lithium‐ion batteries and its capacity retention is about 90% after 1000 cycles.^13^ Additionally, to reduce the cell voltage, FePO_4_, which is prepared by Li^+^ deintercalation from LiFePO_4_, can be used as the cathode, because the reaction of Li^+^ deintercalation and intercalation from/into the LiFePO_4_/FePO_4_ structure is a reversible redox couple (Li^+^ + e + FePO_4_ ↔ LiFePO_4_, *E* ^⊖^ = 0.45 V (vs SHE)), so the theoretical cell voltage of the cell LiFePO_4_/FePO_4_ is zero.

**Figure 1:**
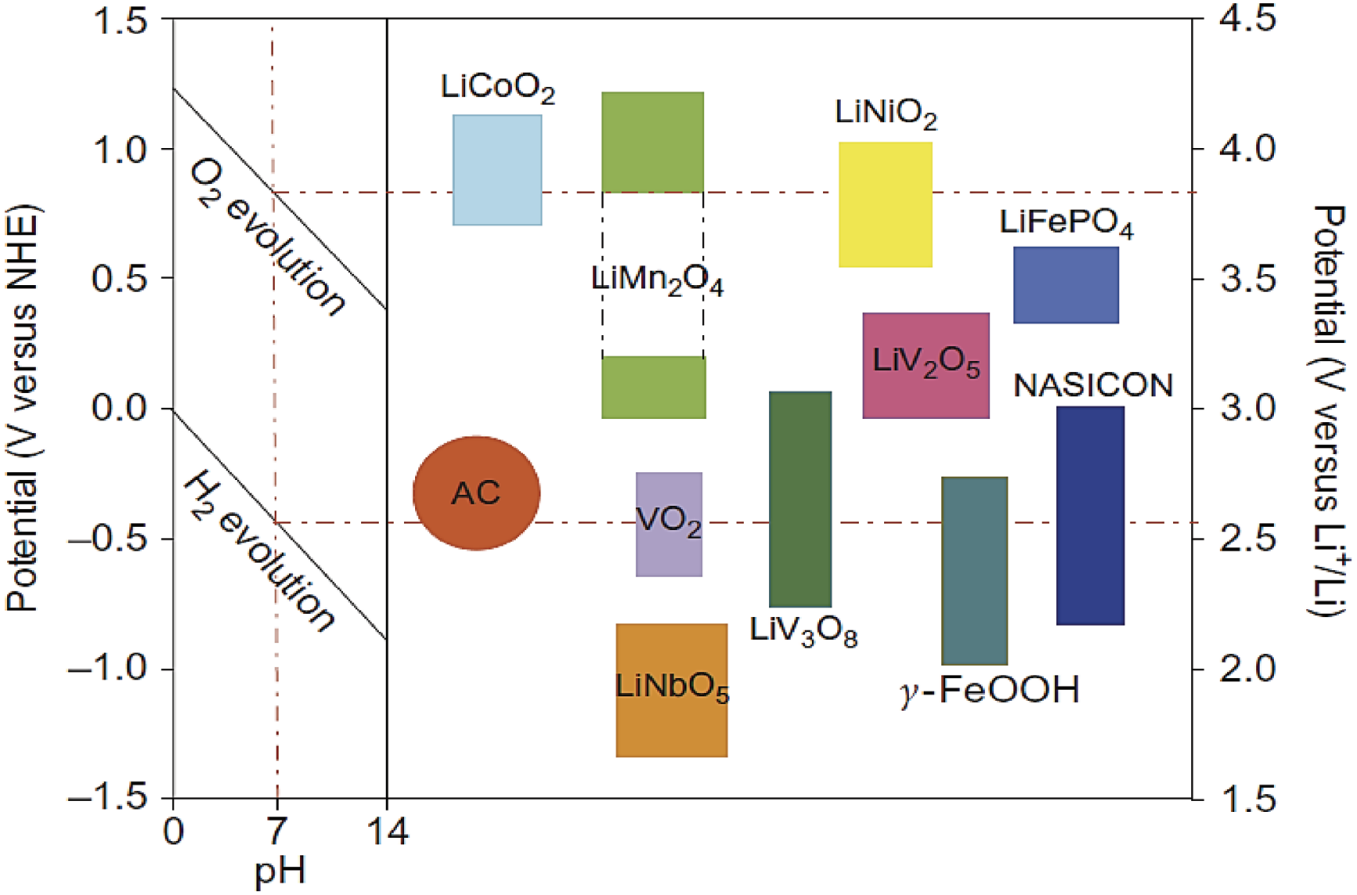
The intercalation potential of some electrode materials that can be used for aqueous lithium‐ion batteries. Left: O_2_/H_2_ evolution potential versus normal hydrogen electrode (NHE) for different pH in 1 mol L^−1^ Li_2_SO_4_ aqueous solution. Right: Lithium‐ion intercalation potential of various electrode materials versus NHE and Li/Li^+^. From Luo et al.^13^

The schematic of the electrolytic cell for lithium extraction from brines is shown in **Figure** **2**. Based on this electrochemical system of “LiFePO_4_ (anode) | supporting electrolyte | anion‐selective membrane | brine | FePO_4_ (cathode),” Li^+^ can be selectively absorbed from the brine in the cathode chamber (Li^+^ + e + FePO_4_ = LiFePO_4_); meanwhile, Li^+^ is desorbed to the anode chamber from LiFePO_4_ (LiFePO_4_ − e = Li^+^ + FePO_4_). The anion‐exchange membrane prohibits the passage of Li^+^ from the anode chamber to the cathode chamber, so Li^+^ can be enriched in the supporting electrolyte. At the end of a cycle, exchanging the negative and positive electrodes and then restarting the electrolytic process, lithium can be extracted continually from the brine and be enriched in the anolyte (filled with a supporting electrolyte).

**Figure 2:**
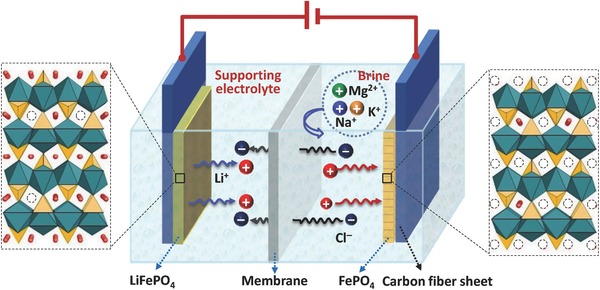
Structure of the electrolytic cell for lithium extraction.

Different from the traditional electrolytes of Li‐ion batteries (in nonaqueous lithium‐ion batteries, the electrolyte is usually an LiPF_6_ solution with a mixture of ethylene carbonate, diethyl carbonate, and dimethyl carbonate; and for the aqueous lithium‐ion batteries, it is always a pure lithium salt solution, such as LiNO_3_, Li_2_SO_4_, and LiCl); however, salt‐lake brines contain much higher concentrations of impurities like Na^+^, Mg^2+^, and K^+^. Therefore, to verify the selectivity of LiFePO_4_/FePO_4_ electrolytic cell, cyclic voltammograms (CV) of LiFePO_4_ were measured in LiCl 0.5 mol L^−1^, NaCl 0.5 mol L^−1^, KCl 0.5 mol L^−1^, MgCl_2_ 0.5 mol L^−1^ solutions, respectively (**Figure** **3**). It can be seen that only one redox couple was found at 0.27 and −0.04 V (vs SCE) in the 0.5 mol L^−1^ LiCl solution, and it corresponded to the de‐intercalation and intercalation of Li^+^ ion from/into the spinel LiFePO_4_/FePO_4_ structure (Figure 3a). While in the 0.5 mol L^−1^ NaCl solution, except an anodic peak situated at 0.29 V (vs saturated calomel electrode (SCE)) which matched the de‐intercalation of Li^+^ from LiFePO_4_ structure in the first anodic sweep process, the other redox peaks reflected the de‐intercalation/intercalation of Na^+^ from/into the NaFePO_4_/FePO_4_ structure (Figure 3b). In the cases of 0.5 mol L^−1^ KCl and 0.5 mol L^−1^ MgCl solutions, except an anodic peak of Li^+^ de‐intercalation from LiFePO_4_ was found, no K^+^ and Mg^2+^ de‐intercalation and intercalation peaks were discovered (Figure 3c,d).

**Figure 3:**
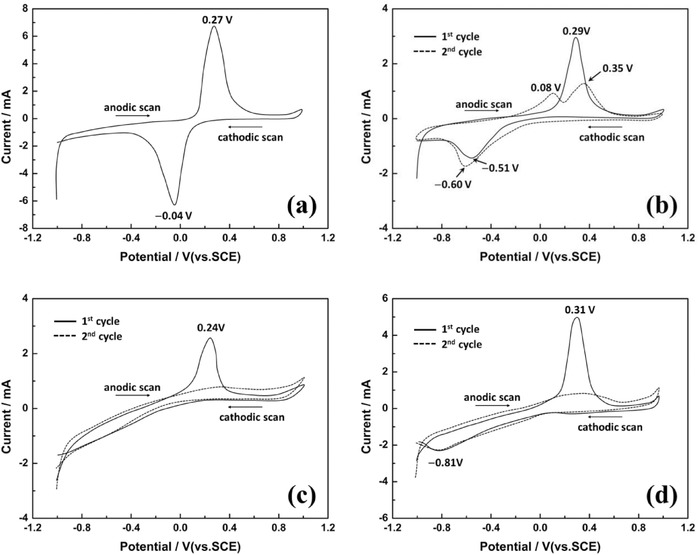
Cyclic voltammograms of LiFePO_4_ in a) 0.5 mol L^−1^ LiCl, b) 0.5 mol L^−1^ NaCl, c) 0.5 mol L^−1^ KCl, and d) 0.5 mol L^−1^ MgCl_2_ solutions.

Comparison with the intercalation behaviors of Li^+^, Na^+^, K^+^, and Mg^2+^, it can be seen that K^+^ and Mg^2+^ almost cannot insert into the FePO_4_ structure and Na^+^ is much more difficult than Li^+^, because the cathodic intercalation potential of Na^+^ is much lower than that of Li^+^. This phenomenon just reveals the embodiment of the ion‐sieve effect, i.e., (1) FePO_4_ is prepared from LiFePO_4_ by an Li^+^ deintercalation process, so the vacant site of FePO_4_ is just suitable for the cation whose ionic radius is close to that of Li^+^. The ionic radii of Na^+^ (1.02 Å) and K^+^ (1.38 Å) are bigger than that of Li^+^ (0.76 Å), so it is difficult for them to insert. (2) Although ionic radius of Mg^2+^ (0.72 Å) is smaller than that of Li^+^, the vacant sites are fit for the monovalent cation, so Mg^2+^ will suffer due to too strong Coulomb repulsion to insert into it.

To further investigate the different intercalation and deintercalation behaviors between Li^+^ and the impurity ions, chronopotentiometry was used (**Figure** **4**). Consistent with the results of cyclic voltammetry shown in Figure 3, FePO_4_ shows a high absorption selectivity for lithium ions and the anodic intercalation sequence is Li^+^ >> Na^+^ > K^+^ > Mg^2+^ (Figure 4a). Conversely, the cathodic deintercalation sequence is Li^+^ < Na^+^ < K^+^ < Mg^2+^ (Figure 4b). In fact, it has a positive effect on the cycling performance, because if some impurity ions are unfortunately intercalated into the FePO_4_ structure, it can be easier to be de‐intercalated from the structure, rather than block the channel and hinder the movement of Li^+^ in LiFePO_4_ and/or FePO_4_.

**Figure 4:**
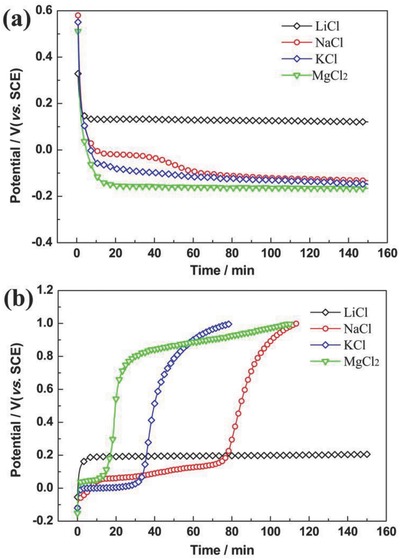
Chronopotentiometric curves of the deintercalation and intercalation of Li^+^, Na^+^, K^+^, and Mg^2+^ ions into/from FePO_4_/LiFePO_4_. a) Cathodic intercalation chronopotentiometric curves; b) Anodic deintercalation chronopotentiometric curves. The current density is 0.2 mA cm^−2^, and the concentration of LiCl, NaCl, KCl, and MgCl_2_ solution is 1.0 mol L^−1^.

To investigate the feasibility of this new lithium recovery method, the Yiliping salt lake brine (chemical compositions Li 97.5 mg L^−1^, Mg 13.1 g L^−1^, K 3.5 g L^−1^, Na 100.4 g L^−1^, Mg/Li = 134.4) was treated via an electrochemical separation system “LiFePO_4_ (anode) | NaCl | anion‐selective membrane | Yiliping brine | FePO_4_ (cathode),” and the results are shown in **Figure** **5**. It can be seen that the recovery ratio of lithium increased with the electrolysis time, and it was raised to about 83% after four cycles (Figure 5a). The lithium concentration in the brine was reduced to about 18 mg L^−1^; meanwhile, its concentration in the anolyte was enriched to about 80 mg L^−1^. Especially, the Mg/Li ratio was decreased from the initial 134.4:1 in the brine to 1.2:1 in the anolyte. In addition, for each cycle, the current density decreased with the increasing electrolysis time (Figure 5b), and it can be ascribed to the increased ratio of lithium intercalation and deintercalation into/from FePO_4_/LiFePO_4_.

**Figure 5:**
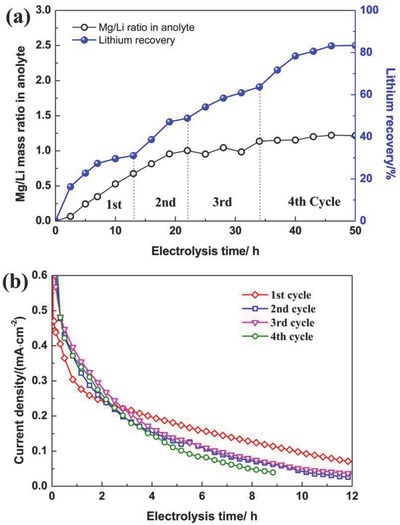
a) Lithium recovery and Mg/Li mass ratio of the anolyte; b) Current density for each cycle.

Based on the experiences shown in Figure 5, a membrane‐stacked electrolytic bath was designed for the extraction of lithium from an old brine solution of China West Taijinar (Li 0.51 g L^−1^, Mg 24.7 g L^−1^, Mg/Li = 48.4), and the device and results are shown in **Figure** **6**. It can be seen that the lithium concentration in the anolyte increased with the cycling, and it reached to about 3.2 g L^−1^ after eight cycles (lithium was enriched about six times). In addition, the Mg/Li ratio was almost maintained at 0.5–0.6:1, which was much lower than that in the initial old brine solution at 48.4:1. Obviously, the new method expressed an excellent characteristic for Mg/Li separation and lithium enrichment.

**Figure 6:**
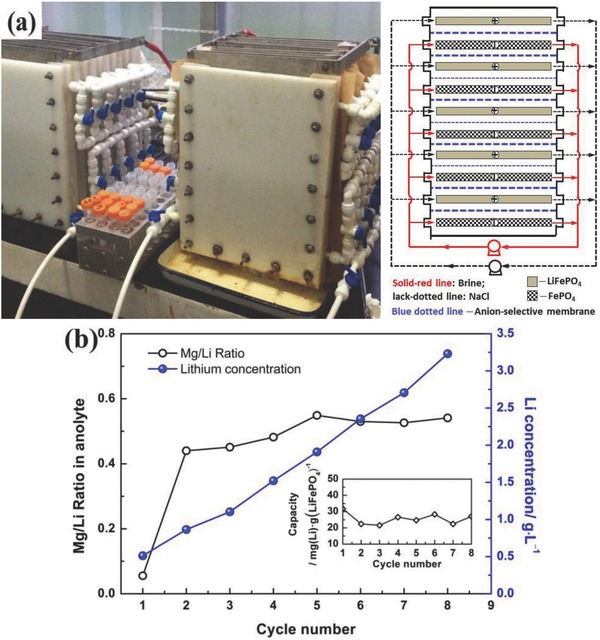
a) Membrane‐stacked electrolytic bath and b) its performance for lithium extraction from the old brine solution (Li 0.51 g L^−1^, Mg 24.7 g L^−1^, K 5.4 g L^−1^, Na 54.6 g L^−1^, Mg/Li = 48.4).

**Figure** **7** shows the cycling performance of the lithium extraction system in a mixed electrolyte of Li^+^ 2 g L^−1^, Mg^2+^ 40 g L^−1^, NaCl 116 g L^−1^ under the conditions of current density 0.3 mA cm^−2^, cutoff value 0.2 V. For the first cycle, the absorption capacity of lithium was about 32 mg (Li) g (LiFePO_4_)^−1^, and ≈84% of the maximal absorption capacity was maintained after 50 cycles. In addition, the cell voltage platform was about 0.05 V for each cycle; it was closed to the theoretical cell voltage of 0 V. Meanwhile, we also noticed that lithium absorption capacity faded slightly. We speculated that the reason can be attributed to the swelling and degumming of electrodes. Fortunately, this capacity fading can be ameliorated by adjusting the composition, i.e., the mixing ratio of LiFePO_4_, carbon black, and polyvinylidene fluoride (PVDF), to enhance the agglutinative property of the particle‐to‐particle and particle‐to‐carbon‐fiber cloth.

**Figure 7:**
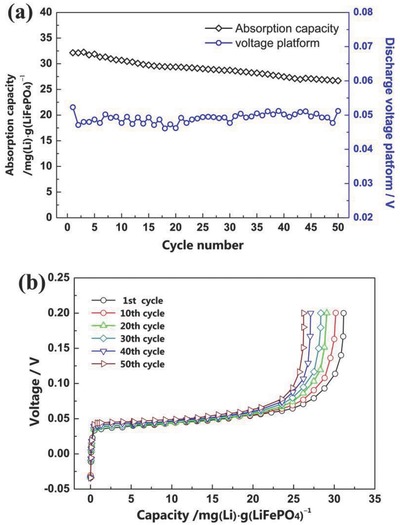
Cycling performance of the electrolytic cell for lithium extraction in a mixed electrolyte of Li^+^ 2 g L^−1^, Mg^2+^ 40 g L^−1^, NaCl 116 g L^−1^.

In summary, an electrochemical system of “LiFePO_4_ (anode) | supporting electrolyte | anion‐selective membrane | brine | FePO_4_ (cathode)” was constructed for selective extraction and concentration of lithium from the brine by using the Li‐ion battery materials. It is a promising technology for lithium recovery from solutions in light of its high selectively, environmental friendliness, and low cost (inexpensive materials and easy assembly), and it avoids the use of poisonous organic reagents and harmful acid or oxidant. In addition, 83% lithium was recovered for the Yiliping brine by this method, with the Mg/Li ratio decreasing from the initial 134.4:1 in the brine to 1.2:1 in the anolyte. For the treatment of the old brine solution from West Taijinar, the Mg/Li ratio decreased from the initial 48.4:1 in the brine to 0.5:1 in the enrichment solution, and the concentration of lithium increased to about six times, corresponding with the lithium absorption capacity of about 25 mg (Li) g (LiFePO_4_)^−1^. More importantly, this approach provides a new dimension to establish the other viable, practical, or more efficient electrochemical separation systems for lithium extraction by selecting more suitable battery materials as the positive and negative electrodes.

## Experimental Section

*Preparation of LiFePO_4_/FePO_4_ Electrodes*: LiFePO_4_ electrode was prepared as follows: (1) mixed LiFeO_4_/C, carbon black, and PVDF thoroughly in *N*‐methyl‐2‐pyrrolidone at the mass ratio of 8:1:1; (2) coated the prepared mixture onto a carbon fiber cloth (material density was about 40 mg (LiFePO_4_) cm^−2^); and (3) dried the electrode in a vacuum oven at 100 °C for 10 h. FePO_4_ was prepared by using the prepared LiFePO_4_ and carbon fiber cloth as the anode and the cathode, respectively. Both of them were placed into an electrolytic cell, which was filled with 0.5 mol L^−1^ NaCl solution, and a constant electrolysis potential of 1.0 V was applied until the current density was less than 0.05 mA cm^−2^.

*Experiments for Lithium Extraction from Brines*: The prepared LiFePO_4_ and FePO_4_ electrodes were used as the cathode and the anode, respectively, IONAC MA‐3475 was used as the anion‐selective membrane. For the extraction of lithium from the Yiliping raw brine solution (chemical compositions Li 97.5 mg L^−1^, Mg 13.1 g L^−1^, K 3.5 g L^−1^, Na 100.4 g L^−1^, Mg/Li = 134.4), the size of both electrodes was 6 × 6 cm^2^. The cathode chamber and the anode chamber were filled with 600 mL of the brine solution and 600 mL 0.5 mol L^−1^ NaCl (supporting electrolyte). For the extraction of lithium from an old brine solution of the China West Taijinar salt lake (chemical compositions Li 0.51 g L^−1^, Mg 24.7 g L^−1^, K 5.4 g L^−1^, Na 54.6 g L^−1^, Mg/Li = 48.4), the size of both electrodes was 28 × 16 cm^2^, and the membrane‐stacked electrolytic bath comprised five pieces of LiFePO_4_ and five pieces of FePO_4_ electrodes. The volume of the brine and NaCl solutions was the same (5.5 L). The separation process was carried out under a constant cell voltage of 0.2 V until the current density reached to the cutoff value of 0.05 mA cm^−2^. At the end of each cycle, the negative and positive electrodes were exchanged and then the electrolytic process was restarted. All the experiences were performed at room temperature.

*Cycling Performance*: The lithium extraction system was tested with Abin instrument BT‐2000 in a mixed electrolyte of Li^+^ 2 g L^−1^, Mg^2+^ 40 g L^−1^, NaCl 116 g L^−1^ under the conditions of current density 0.3 mA cm^−2^, cutoff value 0.2 V. Cyclic voltammetry and chronopotentiometry were carried out with Versa‐STAT4 (Princeton Applied Research, America) at the scanning rate of 0.02 mV s^−1^ and current density of 0.2 mA cm^−2^, respectively. The concentration of ions was determined by ICP‐AES (IRIS intrepid XSP, Thermo Electron Corporation).

## Conflict of Interest

The authors declare no conflict of interest.
